# The role of advocacy and policy in advancing global neurosurgery

**DOI:** 10.1097/MS9.0000000000003052

**Published:** 2025-03-27

**Authors:** Toufik Abdul-Rahman, Sarah M. Badar, Sruthi Ranganathan, Aderinto Nicholas, Joecelyn Kirani Tan, Ogungbemi Evelyn Faith, Mrinmoy Kundu, Andrew Awuah Wireko, Anam Sayed Mushir Ali, Oday Atallah, Viktoriia Horbas, Tetiana P. Teslyk, Valentyna Bumeister

**Affiliations:** a Department of Research, Toufik’s World Medical Association, Sumy, Ukraine;; b Department of Medicine, University of Cambridge, Cambridge, United Kingdom; c Department of Medicine and Surgery, Ladoke Akintola University of Technology, Ogbomoso, Nigeria; d Faculty of Biology, Medicine and Health, University of Manchester, Manchester, United Kingdom; e Institute of Medical Sciences and SUM Hospital, Bhubaneswar, India; f Department of Surgery, Indian Institute of Medical Science and Research, Jalna, India; g Department of Neurosurgery, Carl Von Ossietzky University Oldenburg, Oldenburg, Germany; h Medical Institute, Sumy State University, Sumy, Ukraine

**Keywords:** advocacy, global neurosurgery, low and middle-income countries, neurosurgery, policy

## Abstract

Global neurosurgery has witnessed transformative developments in advocacy and policy aimed at overcoming the barriers that hinder equitable access to neurosurgical care. This paper reveals the stark disparities faced by low- and middle-income countries (LMICs) with an annual burden of 5 million affected individuals. Despite these challenges, the emergence of global neurosurgery stands as a beacon of hope, aspiring to deliver timely, safe, and affordable neurosurgical care. Advocacy and policy play pivotal roles in this endeavour, exemplified by initiatives, like National Surgical, Obstetrics, and Anesthesia Plans (NSOAPs) and Global Neurosurgery Initiatives (GNI), addressing accessibility, training, and disparities. Collaborations between diverse entities and interdisciplinary approaches gain prominence, fostering comprehensive advocacy and policy frameworks. A resolute commitment to equity is discernible, propelling policies toward universal access to neurosurgical care. However, crucial challenges, such as limited resources, awareness gaps, complex political landscapes, data deficiencies, and insufficient international collaborations, must be addressed to see the full potential of these initiatives. While challenges persist, progress is evident through collaborative efforts, technological advancements, and evolving policy landscapes, promising a trajectory toward accessible, safe, and affordable neurosurgical care for all.

## Introduction

Every year, approximately 5 million people in low and middle-income countries (LMIC) are disproportionately affected by neurosurgical conditions, compared to their higher-income country (HIC) counterparts^[1]^, due to limited resources, creating a lack of access to neurosurgical care. This nuanced issue contributes to a vicious cycle, leaving neurotrauma as one of the world’s leading causes of morbidity and mortality worldwide^[2]^. However, the efforts of global neurosurgery have given hope that these pitfalls in neurosurgical care can be alleviated worldwide. Global neurosurgery aims to ensure that timely, safe, and affordable neurosurgical care can be provided regardless of location or economic status^[2]^.

A new-found area of interest in global health is global neurosurgery. The burdens of neurological conditions, such as anencephaly and spina bifida, have sparked the introduction of efforts for global neurosurgery. An example is Public health campaigns to increase food enrichment^[3]^. This, in turn, addresses economic barriers that contribute to financial losses associated with neurotrauma^[3]^.

With this in mind, it raises the question, what has allowed global neurosurgery to maintain its stance, and how can this continue to advance globally? Out of a global estimated 234 million surgeries, LMICs only receive 3.5%^[4]^. Achieving universal health care undoubtedly goes beyond neurosurgeons; it requires collaborative efforts, as this can help proactively address the poorer outcomes of neurosurgical care in LMICs^[5]^. For instance, existing research recognizes that the country-driven National Surgical, Obstetrics, and Anesthesia Plans (NSOAPs), developed mainly through active collaborations, ranging from partners to institutions, remains one of the most distinguished policies integrated, due to its capabilities to address critical issues, foster collaborations, and streamline health services, and when used to its full potential, can overcome existing barriers in LMICs^[6]^.

Other policies implemented to tackle these pressing limitations in global neurosurgery include the works of Harvard Medical School’s Global Neurosurgery Initiative (GNI) establishing collaborative partnerships within LMICs such as providing ongoing pediatric neurosurgical training in Uganda^[7]^.

These unique approaches to integrate surgical care into national health plans suggest that neurosurgical care can thrive through the implementation of strategies, such as policies and advocacy efforts. Advocacy and policy go hand-in-hand to effectively push change, with advocacy referring to a means of promoting policies to enhance health equity; whereas policy is fixated on the formulation and dissemination of regulations and plans to achieve societal public health goals^[8,9]^. Thus, by coupling policy with advocacy, governments and organizations can promote awareness, funding, and access to neurosurgical care, especially in LMICs, where the burden of neurological diseases is the highest globally^[3]^.

Advocacy and policy are of significant value toward improving neurosurgical outcomes. Thus, the role of this narrative review is to critically explore the factors contributing to advancing global neurosurgery, and address the existing barriers and recent innovations that can potentially be adopted in practice to further enhance the neurosurgical field.

## Methods

This narrative review employed a rigorous methodology to explore the role of advocacy and policy in advancing global neurosurgery (Table 1). The review was conducted with a focus on identifying relevant studies, policies, and initiatives that address the disparities in neurosurgical care, particularly in LMICs. We performed a comprehensive search of three major databases: PubMed, Scopus, and Google Scholar. The search strategy incorporated a combination of keywords and phrases, such as “global neurosurgery,” “neurosurgery,” “advocacy,” “policy,” “LMICs,” “Low and middle-income countries,” “National Surgical, Obstetrics, and Anesthesia Plans,” and “Global Neurosurgery Initiative.” Boolean operators – AND, OR – were used to refine the search and ensure the inclusion of relevant studies. In addition to database searches, a manual review of references cited in selected articles was performed to identify additional relevant studies.

**Table 1:** Summary Table of methodology for this narrative review

Methodology steps	Description
Literature search	PubMed, Scopus, and Google Scholar
Inclusion criteria	Studies published in English between 2000 and 2024 Various study designs, such as experimental, cohort, case-control, cross-sectional, and descriptive studies Studies that focused on the role of advocacy and policy in neurosurgical care Studies that addressed neurosurgical disparities in LMICs Studies that discussed the implementation and outcomes of policies, such as NSOAPs or similar initiatives
Exclusion criteria	Articles unrelated to the intersection of advocacy, policy, and neurosurgery Articles that focused solely on high-income countries without discussing LMIC implications Unpublished studies Standalone abstracts.
Search terms	“global neurosurgery”, “neurosurgery”, “advocacy” “policy”, “LMICs”, “Low and middle-income countries,” “National Surgical, Obstetrics, and Anesthesia Plans,” and “Global Neurosurgery Initiative”. Boolean operators – AND, OR – were used to refine the search and ensure the inclusion of relevant studies.
Additional search	A thorough inspection of references mentioned in selected studies through manual examination No predefined restriction on the number of studies to be considered

The inclusion criteria for this review encompassed various study designs, such as experimental, cohort, case-control, cross-sectional, and descriptive studies, that focused on the role of advocacy and policy in neurosurgical care, addressed neurosurgical disparities in LMICs, discussed the implementation and outcomes of policies, such as National Surgical, Obstetrics, and Anesthesia Plans or similar initiatives, and were published in English between 2000 and 2024 to capture the most relevant and recent developments in global neurosurgery. Articles were excluded if they were unrelated to the intersection of advocacy, policy, and neurosurgery, focused solely on high-income countries without discussing LMIC implications, or were unpublished studies or standalone abstracts. Key data points were extracted from the selected studies, including the geographic focus of the study or initiative, the type of policy or advocacy effort discussed, the reported outcomes or impacts on neurosurgical care, and challenges and barriers identified in the implementation of advocacy or policy initiatives. The extracted data were synthesized into a narrative format to provide a critical assessment of the role of advocacy and policy in global neurosurgery. This synthesis also highlighted innovative approaches and best practices that could inform future initiatives.

## Results and discussion

### Discussing the recent developments in advocacy and policy in global neurosurgery

####  

##### Advocacy

**Figure 1.**
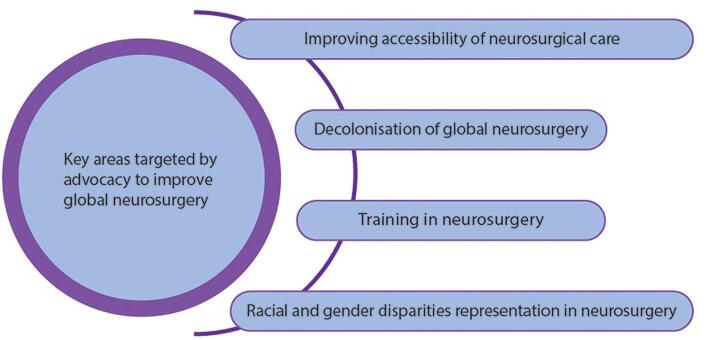
Key areas targeted by advocacy to improve global neurosurgery.

Advocacy plays a key role in advancing global neurosurgery, with successful campaigns or efforts including the Neurology and Neurosurgery Interest Group (NANSIG), the Global Alliance for Prevention of Spina Bifida-F (GAPSBiF), Intersectoral Global Action Plan (IGAP), and organizations such as Congress of Neurological Surgeons (CNS)^[10]^. These noteworthy efforts in global neurosurgery promotion often focus on four main areas: (i) improving accessibility of neurosurgical care, (ii) training in neurosurgery, (iii) racial and gender disparities in representation in neurosurgery, and (iv) decolonization of global neurosurgery, which is part of the larger movement to decolonize global health, and involves prioritising equity promotion in healthcare (Fig. 1)^[11]^. Improving accessibility of neurosurgical care can be further categorised as: (i) initiatives to improve affordability and the utilization of neurosurgical care and (ii) initiatives to improve the availability of neurosurgical care. To address the high costs involved in neurosurgery, the Global Neurosurgical Committee (GNC) has been involved in advocating the inclusion of neurosurgical care within Universal Health Coverage^[12]^.

Insurance limitations have been shown to affect the utilization of neurosurgical care^[13]^. In some countries, particularly LMICs, insurance can be poorly established or underutilized, causing heavy reliance on personal funds for healthcare^[14]^. Further exacerbated by the lower income in some LMICs, patients can refuse neurosurgical interventions simply due to the cost. A study estimated that the cost of neurosurgical procedures can vary between $291 to $1221 in a low-resource setting, and given that many individuals often rely on less than $6 a day, this cost is exceptionally high to bear^[15,16]^. Therefore, addressing the affordability of neurosurgical interventions by advocating better-established insurance programs – such as the effort by GNC – forms a key pillar of advancing global neurosurgery. Beyond improving affordability, improving access to neurosurgical care involves improving the availability of these interventions. Due to a wide range of reasons, such as a lack of personnel, facilities, and equipment, there can be limitations in the availability of neurosurgical care provision in LMICs^[17,18]^. For instance, though the overall number of neurosurgeons is estimated to be 50 000, with the unequal distribution of these experts throughout the regions, low-resource countries are reported to have 0.01–0.1 neurosurgeons per 100 000 people^[19]^. This is further exacerbated by other limitations in resources, such as electricity due to power cuts.

Efforts to train neurosurgical staff include the collaboration between Duke University’s neurosurgery department and Mulago Hospital in Kenya^[20]^, and the establishment of neurosurgical residency programs in Haiti^[21]^. These initiatives are supported by advocacy efforts, such as the Global Action Plan by the World Federation of Neurosurgical Societies (WFNS) GNC^[3]^. Advocacy efforts also include some support for the controversial initiatives to train non-doctors as neurosurgeons in sub-Saharan Africa^[22]^. Given that the availability of skilled healthcare professionals is especially low in certain countries, as seen via the low ratio of staff per 100 000 patients^[23]^, efforts to promote awareness of this issue and training of these personnel are important in improving neurosurgical care in an equitable manner.

In addition to training neurosurgical staff, there are advocacy efforts to reduce racial and gender disparities in representation in neurosurgery. Given that the majority of neurosurgeons are male^[24]^, potentially due to the long, arduous training in neurosurgery, lack of mentorship opportunities, societal pressures, and concerns regarding discrimination and harassment^[25,26]^, females are less represented in neurosurgical care teams. In addition, racial disparities in neurosurgery are reported, where neurosurgical residency programs show the greatest differences between the proportion of Black/African-American residency applicants and current residents^[27]^. Since representation can impact healthcare outcomes^[28]^, the push for more female neurosurgeons and neurosurgeons from diverse ethnic and racial backgrounds is a key goal in global neurosurgery. NANSIG forms a noteworthy voice for gender disparities in neurosurgical representation^[29]^, while physicians, such as Drs. Nnenna Mbabuike and William W. Ashley Jr, have shown involvement in alleviating racial disparities^[30]^.

Lastly, the decolonisation of global neurosurgery is part of the larger, recent movement to “decolonise global health”^[31–34]^, which aims to address the “neocolonial patterns of relations in medicine”^[3]^. Advocates believe that this movement is key to eliminating the hierarchical structure and disparities between and within countries. Decolonization of global neurosurgery (or global health, more broadly) is proposed to cause shifts in the power dynamics in the global system that can drive the development of more equitable policies and initiatives^[34]^. Advocating global neurosurgery will lead to the development of efforts that meet high ethical standards, and destabilize inequitable, hierarchical systems^[3]^. Recognizing the importance of this movement, the WFNS GNC (2021–2023) has a designated Decolonization Lead.

Beyond these common themes, successful advocacy strategies identified for the promotion of global neurosurgery include the organization of neurosurgeons into larger groups to have a larger voice, the implementation of neurosurgical health campaigns tailored to common neurosurgical conditions in various regions, execution of equitable epidemiologic and economic studies to better understand neurosurgical conditions to support public health efforts, establishment of nonprofit foundations, and contribution in management or policy-making efforts^[3]^.

Two pertinent examples of advocacy efforts in the field of global neurosurgery, GAPSBiF, and NANSIG (Table 2), are further detailed. GAPSBiF involves the coordinated efforts of experts from various fields such as “neurosurgeons, paediatricians, geneticists, epidemiologists, food scientists, and fortification policy makers or stakeholders”^[35]^, to advocate for universal folic acid fortification (FAF) of staple foods, primarily to prevent the prevalence of conditions, such as spina bifida. In addition, the team involves students to bolster social media efforts to engage with the general public. These international efforts are also bolstered by national advocacy efforts, seen in countries, such as Costa Rica and Ethiopia^[3]^. As part of strengthening and justifying their advocacy efforts, GAPSBiF also engages in research and produces abstracts and manuscripts in high-impact journals to establish the importance of food fortification. NANSIG is an organization that aims to inspire the next generation of neurosurgeons^[29]^. They initiated an advocacy campaign in 2020 to illuminate the challenges and motivations of individuals in neurosurgery, with a focus on how these might be different for women. This campaign, “Neurosurgeon of the Month” has been reported to achieve success in motivating more female medical students to consider careers in neurosurgery, inspired by current role models^[29]^.

**Table 2:** A concise overview of how each advocacy intervention aligns with the criteria of being evidence-based, ethical, empowering, and collaborative within the public health advocacy assembly line framework

Advocacy Intervention	Evidence Based?	Ethical?	Empowering?	Collaborative?
Neurology and Neurosurgery Interest Group (NANSIG)	Yes	Yes	Yes	Yes
Global Alliance for Prevention of Spina Bifida-F (GAPSBiF)	Yes	Yes	Yes	Yes
Intersectoral Global Action Plan (IGAP)	Yes	Yes	Yes	Yes
Congress of Neurological Surgeons (CNS)	Yes	Yes	Yes	Yes
Global Neurosurgical Committee (GNC)	Yes	Yes	Yes	Yes
Duke University’s neurosurgery department and Mulago Hospital collaboration	Yes	Yes	Yes	Yes
Establishment of neurosurgical residency programs in Haiti	Yes	Yes	Yes	Yes
Advocacy for the inclusion of neurosurgical care within Universal Health Coverage	Yes	Yes	Yes	Yes
GAPSBiF and Folic Acid Fortification as a Targeted Neurosurgical WHA Resolution	Yes	Yes	Yes	Yes
Advocacy for training non-doctors as neurosurgeons in sub-Saharan Africa	Yes	Yes	Yes	Yes
Advocacy efforts by physicians Drs. Nnenna Mbabuike and William W. Ashley Jr	Yes	Yes	Yes	Yes

##### Policy

Other than advocacy, policies form a key component of efforts to advance the goals of global neurosurgery. Major goals of global neurosurgery often addressed by policies include: (i) improving healthcare outcomes for a few key neurological conditions and (ii) improving the affordability of neurosurgical care.

Neurosurgical conditions that disproportionately affect individuals globally, with a higher incidence in LMICs include traumatic brain injury, spina bifida, and spinal cord injury^[36-38]^. Policies to address such conditions include the NSOAPs and Comprehensive Policy Recommendations for the Management of Spina Bifida and Hydrocephalus in LMICs^[39]^. Since the lack of essential micronutrients is an important causative factor in spina bifida, efforts to address it also include policies to address the underlying reasons behind the lack of essential micronutrients in some regions. This includes the national food fortification programs following the coordinated efforts between policymakers and neurosurgeons in Ethiopia^[22]^, addressing the disproportionate incidence of spina bifida in LMICs. The implementation of these programmes has also been successfully applied in countries, such as Jordan and Lebanon^[40]^ through enforcing national flour programmes, this has thus helped give rise to a 2.4.% annual reduction across countries for the preponderance of anaemia in non-pregnant women as identified by Barkley, *et al*^[41]^.

Road and safety management is key pillar to addressing traumatic brain injury. examples of policy frameworks on it include Vision Zero in Sweden and the formation of the National Council for Road Safety in France^[3]^. Utilization of such councils is undoubtedly pivotal towards reducing preventable neurosurgical cases. For instance, France’s efforts have complied over time contributing to their recognition as the 12th out of 27 EU countries in terms of the lowest numbers of fatalities per million inhabitants^[42]^. Thus, although given the longstanding complexities in terms of public and national perspective, implementation and design^[43]^ posed a major challenge in achieving decade-goal lessons for the next span. Perhaps this could be a factor to be encouraged and examined more closely under the microscope within many LMICs, as more links are built between these two classes.

Since high costs are a key barrier to accessing neurosurgical interventions, policies for better insurance coverage are important to advancing global neurosurgery. This includes World Health Assembly (WHA) Resolutions 68.15 and 72.31^[2]^. Other noteworthy examples of policies to advance global neurosurgery include both international and national policies. International policies can include those indirectly resulting from the United Nations Sustainable Development Goals (SDGs). Since 14 of the 17 SDGs are relevant to neurosurgery, efforts to develop policies that promote the attainment of these goals advance global neurosurgery^[44]^. More examples of policies at the international level include food fortification efforts in Ethiopia, as discussed previously, the Comprehensive Policy Recommendations for the Management of Spina Bifida and Hydrocephalus in Low- and Middle-Income Countries^[39]^, which can be categorized into six sections involving pre-hospital and outpatient care, surgical systems, rehabilitation services, long-term care services, and public health efforts^[45]^. Such actions have helped to promote the hope that from a governmental standpoint, through policy and advocation working in synchronization, 80% of a given population can be within 2 h of spina bifida care^[36]^.

However, on the contrary, it is also important to note that the use of such policies or programmes without proper medical evaluation of population intakes has led to opposite effects, leading to patient dissatisfaction. A prime example of this is the introduction of Vitamin A programmes in LMIC, including Malawi and South Africa, where layering of these programmes has been found to contribute to hypervitaminosis A^[46]^ as patients are provided excess micronutrients.

Lastly, it is also noteworthy that advocacy efforts can influence policies. This is seen in countries such as Costa Rica and Ethiopia^[3]^. Recently, the Ethiopian government made it compulsory to fortify wheat and cooking oil, and it is believed that advocacy by neurosurgeons was essential in spearheading this policy implementation movement^[13]^.

#### Barriers to effective advocacy and policy in global neurosurgery

**Figure 2.**
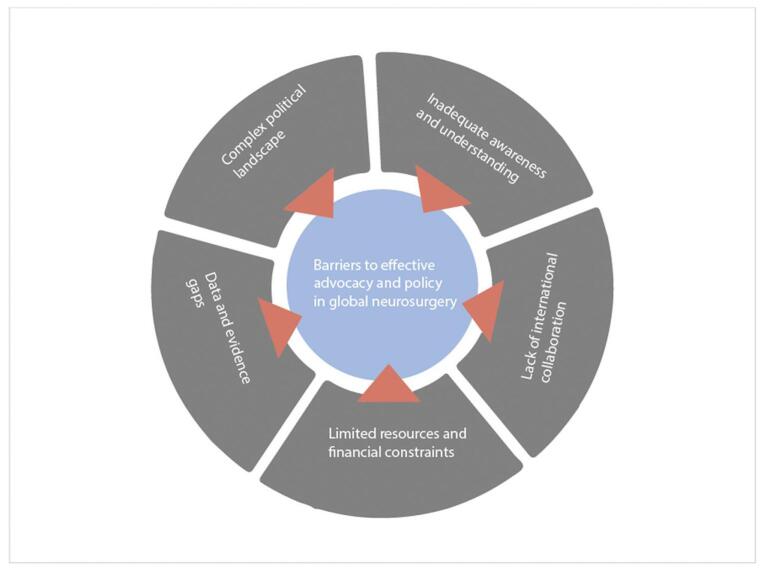
Barriers to effective advocacy and policy in global neurosurgery.

The advancement of global neurosurgery is plagued by several challenges, presenting formidable barriers to effective advocacy and policy implementation (Fig. 2)

##### Limited resources and financial constraints

The quest for fair healthcare in global neurosurgery relies on sufficient financial support^[47]^. This challenge is particularly severe in regions with limited economic resources, posing a major challenge to effective advocacy and developing policies to enhance neurosurgical care worldwide^[47]^. This impact is most deeply felt in areas with low economic indicators^[1]^. These regions face numerous developmental challenges and must distribute their limited resources among various pressing needs, such as education, infrastructure, and public health^[1]^. Allocating enough funds for healthcare initiatives, especially neurosurgery, becomes exceptionally challenging in financially constrained contexts. Neurosurgical care demands a comprehensive system with advanced infrastructure, sophisticated equipment, well-trained medical professionals, and a consistent supply of essential medical resources^[48]^.

For advocates and policymakers in global neurosurgery, the lack of financial resources presents a multifaceted challenge. Effecting meaningful changes requires navigating a complex landscape of budget negotiations, competing priorities, and strategic resource allocation^[21]^. Advocacy efforts must highlight the far-reaching consequences of limited funding for neurosurgical care, emphasizing how it affects the patient outcomes, public health, and overall societal well-being. This requires an approach grounded in robust evidence, combining compelling narratives, empirical data, and case studies to underscore the tangible impact of neurosurgical care on the effectiveness of healthcare overall.

This calls for a nuanced strategy that addresses the immediate financial shortfall and strives to establish a sustainable environment for neurosurgical care. This involves advocating for comprehensive reforms in healthcare budgeting, establishing partnerships with international organizations, and promoting the alignment of global health agendas to prioritise neurosurgical interventions.

Beyond a lack of financial resources, LMICs face a lack of good quality infrastructure. WHO reports that 95% of the medical equipment in LMICs is imported but 40% of them cannot be used, compared to only 1% in HICs, due to the lack of staff to maintain the equipment regularly^[19]^. In addition to poor healthcare resources, poor infrastructure in general limits the access to healthcare, which includes neurosurgery. It is estimated that 75.6% of patients need to travel over 2 h to access neurosurgical care compared to 33.6% in HICs. This is particularly problematic when considering the acute presentation in certain neurosurgical pathologies, such as intracranial hemorrhage^[19]^.

##### Inadequate awareness and understanding

The complex nature of neurosurgery remains unfamiliar to the general public and certain segments of healthcare professionals^[49]^. This lack of awareness creates a major obstacle that undermines advocacy efforts, leading to a lack of public support and inadequate discussions on policy decisions related to improving neurosurgical care. Despite its crucial role in public health and treating neurological conditions, there is often a persistent need for more awareness about neurosurgery’s basics, advancements, and potential impact^[50]^. For instance, certain neurosurgical conditions, such as epilepsy, are associated with witchcraft in some countries, such as Ethiopia. Therefore, it is reported that across countries, such as Ethiopia, Kenya, and Nigeria, that religion and culture play a significant role in coping with neurosurgical diagnoses. Patients are, therefore, noted to be less responsive to Western medicine or prefer traditional healing practices^[51]^.

Effective advocacy relies on public support and engagement, but when neurosurgery remains mysterious, the connection between advocates and the public breaks down^[52]^. This results in a situation where the importance of investing in neurosurgical care, potential improvements in patient outcomes, and the broader societal benefits of better neurological health remain unknown to the public. Informed policy decisions require robust discussions involving various perspectives and expert insights^[52]^. However, the lack of awareness leads to a need for more informed discussions about policy matters related to neurosurgical care. Decisions impacting resource allocation, infrastructure development, and research funding may only be made with a full understanding of the implications and potential benefits of investing in neurosurgery. That being so, when options seem so little, what can we as communities do to help close the awareness rift?

A multifaceted approach is necessary to overcome this challenge, involving education, communication, and collaboration. Advocacy efforts should bridge the knowledge gap through targeted public awareness campaigns, sharing knowledge, and collaborating with educational institutions and healthcare organizations. By highlighting the critical role of neurosurgery in improving neurological conditions, quality of life, and societal well-being, advocates can create an environment conducive to informed support and thoughtful policy discussions.

Effective communication is also essential to convey complex concepts to diverse audiences^[53]^. Through clear language, engaging visuals, and relatable stories, advocates can demystify neurosurgery’s complexities and raise awareness. Engaging with media, social media platforms, and public forums can further spread knowledge and stimulate informed conversations. Albeit it’s difficulties in an indisputable growing digital era, a notable idea, could establishing easily accessible resources to build foundational knowledge regarding neurological trauma and alongside guidance on how individuals can play a preventative role where possible. Such concepts are being continuously trialled, for instance, the formation of a vaccine safety network (VSN) has been developed by the WHO and underwent pilot studies to bring conversation around vaccine safety^[54]^, which is known to spark attitudes of hesitancy or negative behaviours in LMIC.

Another factor to consider is that as part of a conscious effort to improve awareness and neurotrauma care in LMICs on a longer-term basis, as highly vouched with by clinical teams there should be “more hospitals, more neurosurgical training centers, and more funds allocated to research”^[55]^.

This can undoubtedly boost awareness and assist in further grasping concepts and these funds can further allow for a continued stream of care for patients, for instance, ideas raised about these funds include that they “should be going into each and every hospital even by the government […] there should be some sort of transportation for these patients. So if transportation is available, I think it will be much easier to follow those patients”^[55]^. This gesture can thus allow patients to have a greater sense of commitment alongside practical means to fully receive adequate care to learn, treat and manage their neurotrauma.

Aside from infrastructural changes, helping to build awareness and education, an another option that can complement but often isn’t fully utilized in LMIC to its fullest potential^[56]^, is the use of social media, which has already shown success in healthcare for disease surveillance, knowledge attainment, and translation alongside mass communication^[57]^.

##### Complex political landscape

The intricate interaction of political forces presents a significant challenge in advocating for global neurosurgery and implementing related policies. The complex web of political dynamics within countries adds an extra layer of complexity that greatly affects the effectiveness of advocacy efforts^[58]^. Those working to advance neurosurgical care often find themselves maneuvering through a complex maze of resistance stemming from competing political priorities, bureaucratic complexities, and the uncertainties introduced by changes in government leadership^[58]^. Pertinent examples include Afghanistan and Iraq where the political instability has impacted the healthcare systems, causing their residents to rely on healthcare services in nearby countries. The existence of political unrest can also impact the allocation of financial resources that can influence the level of care the residents can achieve. It is estimated that the budget for healthcare only makes up 3% of the overall government annual budget in Kenya compared to 15% in the USA^[59]^.

Political agendas encompass a range of priorities, including economic development, public welfare, and security considerations^[60]^. Despite its importance, advocating for neurosurgical care often needs help to gain attention amidst other pressing issues^[58]^. The difficulty arises when the push for progress in neurosurgery competes for recognition and resources against matters that may be seen as more urgent or politically advantageous.

The intricate administrative processes and regulatory frameworks within government systems can significantly hinder the smooth advancement of neurosurgical advocacy and policy development^[61]^. The convoluted procedures, required approvals, and bureaucratic structures can create obstacles that delay translating advocacy goals into concrete policy changes. Navigating these bureaucratic challenges can extend timelines, dampen enthusiasm, and increase the overall complexity of the advocacy process^[61]^.

The fluctuation in government leadership, often triggered by elections or changes in administrative bodies, introduces an element of unpredictability that further complicates advocacy efforts and creates gaps in healthcare governance. Leadership changes can lead to shifts in healthcare priorities, disrupt ongoing policy initiatives, and demand adjustments in advocacy strategies^[62]^. Advocates must adapt to these changes while steadily focusing on advancing neurosurgical care. Maneuvering through the political landscape requires a strategic approach that acknowledges and addresses these inherent challenges. Whilst this can sometimes be frowned upon across both HIC and LMICs, whistleblowing can serve as a powerful anti-corruption tool against health & pharmaceutical companies, proven to inadequately serve communities. Successful observations of this have been seen through the “I Speak Out Now!” Campaign in Malawi encourages individuals to speak out against drug theft which led to increased prosecutions, fines, and arrests for injustice^[63]^. Thus, advocates must understand that political dynamics are not uniform but are primarily shaped by negotiation, influence, and engagement.

##### Data and evidence gaps

The essence of effective advocacy and well-informed policy-making rests upon decisions driven by data^[64]^. Yet, a significant challenge emerges in global neurosurgery: data and evidence gaps. This scarcity of comprehensive and robust data creates a notable barrier that obstructs the creation of knowledgeable policies and weakens the capacity to convincingly illustrate the meaningful contributions of neurosurgical care to public health and societal well-being^[65]^.

Data forms the bedrock upon which successful advocacy and policy choices are constructed^[66]^. It furnishes a factual foundation for recognizing patterns, evaluating needs, and projecting potential outcomes. However, the global neurosurgery landscape is characterized by data gaps due to limited research funding, resource constraints, variations in data collection methodologies, and difficulties in sharing and standardizing data across different regions^[65]^. A study reports that across 80 journals, the neurosurgical research by LMICs remains low^[67]^.

Effective policies require an in-depth comprehension of the present status of neurosurgery, the prevalence of neurological disorders, the influence of interventions, and the attained health outcomes. Without comprehensive data, policymakers operate with restricted insights, which may lead to suboptimal allocation of resources and policy determinations that fall short of addressing critical necessities.

##### Lack of international collaboration

Global neurosurgery is inherently connected to the necessity of international collaboration, especially when addressing challenges that surpass national boundaries and striving to facilitate the exchange of best practices^[68]^. However, this crucial collaboration often encounters a complex mix of logistical, cultural, and regulatory obstacles that can impede the establishment and continuity of effective mechanisms for global teamwork^[68]^.

The intricate nature of neurosurgical care frequently calls for collaboration across borders to confront multifaceted challenges. From sharing advanced surgical methods to addressing global health concerns like neurotrauma and neurological diseases, the importance of international cooperation cannot be underestimated. The worldwide sharing of knowledge, expertise, and resources becomes essential in elevating care standards and enhancing patient outcomes globally.

Efficient international collaboration in neurosurgery can be hindered by logistical hindrances arising from geographical separations, differences in time zones, and technological limitations^[69]^. Coordinating activities, disseminating information, and enabling real-time communication can be challenging when different locations are involved. These logistical obstacles can delay the swift exchange of crucial insights and the timely implementation of collaborative projects. Cultural variations and the diverse professional backgrounds of neurosurgeons globally introduce an added layer of intricacy to international collaboration^[25]^. Differences in communication styles, medical practices, and ethical considerations can lead to misunderstandings or mismatches in collaborative endeavors. Bridging these cultural gaps necessitates sensitivity, effective communication approaches, and mutual appreciation for differing viewpoints.

The regulatory frameworks governing healthcare, medical research, and cross-border partnerships differ significantly from one country to another^[70]^. Diverse ethical standards, research protocols, and legal requisites can complicate establishing collaborative efforts. Navigating these regulatory complexities requires deeply understanding local regulations and reconciling differing regulatory requirements.

### Innovations and emerging trends in advocacy and policy in global neurosurgery

Advocacy and policy-making in global neurosurgery are constantly changing, shaped by the dynamic interplay of healthcare advancements, technological progress, and increasing recognition of the importance of equitable access to neurosurgical care^[1]^. As these elements come together, they give rise to many developments that can reshape how neurosurgical care is advocated for and how policy decisions are formed worldwide.

Efforts to bridge the existing gaps in data have gained significant momentum within this evolving landscape^[1]^. Collaborative endeavors among researchers are gaining traction as they gather comprehensive data about neurosurgical needs, outcomes, and disparities^[71]^. This growing database of information is becoming an essential tool that informs evidence-based advocacy strategies and guides the creation of well-informed policy recommendations^[71]^. The convergence of data-driven insights and advocacy initiatives has the potential to drive impactful changes in the availability and quality of global neurosurgical care.

Alongside data-driven approaches, initiatives aiming to train and empower local healthcare professionals in underserved regions are also gaining prominence. These initiatives encompass a range of activities, including developing training programs, organising workshops, and providing mentorship opportunities^[3]^. By investing in such capacity-building efforts, sustainable neurosurgical capabilities are nurtured within these regions. This approach enhances local expertise and contributes to establishing a self-sustaining healthcare infrastructure that can effectively address neurosurgical needs^[3]^.

The transformative power of technology is evident through the adoption of telemedicine platforms in global neurosurgery^[72]^. These platforms are being utilized to facilitate various activities, from virtual consultations to mentorship and remote surgical guidance^[72]^. By overcoming geographical barriers, these technologies bridge the expertise gap between neurosurgeons in well-resourced environments and patients in underserved regions with limited access to specialized care. However, it is important to note that, particularly within LMICs and resource-limited settings technologies should not further overburden clinical teams and financial circumstances, which needs to be attentively considered in these areas^[55]^.

Furthermore, advocacy and policy in neurosurgery are witnessing an increasing prevalence of collaborations between diverse entities, including institutions, governments, and international organizations^[73]^. These collaborations act as powerful drivers of policy reforms, resource sharing, and collective efforts to elevate the standards of neurosurgical care on a global scale. This collaborative approach underscores the recognition that the intricacies of neurosurgical care go beyond individual domains and require a united effort to bring about meaningful change.

The integration of digital health platforms, mobile applications, and electronic health records holds the potential to revolutionize advocacy and policy development. This seamless integration can streamline data collection, enhance patient management, and optimize resource allocation. Ultimately, it ushers in a new era of efficiency in advocacy initiatives and policy formulation. Concurrently, the advent of AI-powered tools is redefining data analytics. These advanced tools can navigate extensive datasets, uncover hidden trends, predict disease patterns, and optimize resource distribution. This analytical capability translates into targeted advocacy strategies and evidence-based policy recommendations, enhancing the effectiveness of global neurosurgery efforts.

Another key point is the use of technological languages being used to transform the world of neurosurgical care as we know it. For instance, through machine learning, there is a huge potential for it to be used efficiently to help manage neuro-oncological cases to even incidences of aneurysms related to neurotrauma^[74]^. Another critical example includes the use of AI to reduce postoperative facial nerve palsy, such as Bell’s palsy, in parotid surgery^[75]^.

A noteworthy shift in advocacy strategies is the increasing focus on patient narratives and their active involvement in policy discussions. By amplifying patient voices, advocacy efforts gain a deeper emotional resonance and ensure policies are grounded in the needs and experiences of those seeking neurosurgical care. Furthermore, recognizing the diplomatic aspect, governments, international organizations, and stakeholders are embracing collaborative health diplomacy efforts. This recognition underscores the potency of joint diplomatic endeavors in breaking down barriers that hinder resource allocation and impede policy reform. The infusion of health diplomacy adds a fresh layer to global neurosurgery advocacy, transcending geographical and political constraints.

Collaboration is emerging as a driving force in shaping advocacy and policy in neurosurgery. The convergence of neurosurgery with various medical disciplines, public health experts, economists, and social scientists is gaining prominence. This interdisciplinary alliance fosters comprehensive advocacy approaches and policy frameworks, harnessing diverse perspectives’ collective wisdom to address the neurosurgical landscape’s multifaceted challenges. Moreover, a rising trend emphasizes a stronger focus on addressing global health inequalities through neurosurgical advocacy and policy. The recognition of neurosurgery as a crucial component of universal health coverage prompts the reevaluation of policies, urging the prioritization of equitable access to neurosurgical care on a global scale.

### Study limitations

Despite the comprehensive exploration of advocacy and policy roles in advancing global neurosurgery, this study has certain limitations that must be acknowledged. Firstly, the review relied on existing literature, which may have inadvertently excluded relevant studies due to constraints, such as publication language or the selection of specific databases. While efforts were made to incorporate a broad range of sources, the search strategy predominantly focused on English-language publications, introducing potential language bias and limiting perspectives from non-English speaking regions. Also, the availability and quality of data on advocacy and policy initiatives in LMICs remain a significant challenge. Data deficiencies, inconsistencies in reporting, and limited access to detailed accounts of program implementation may have impacted the depth and comprehensiveness of the analysis. Furthermore, disparities in the reporting of neurosurgical outcomes between LMICs and HICs may have led to an incomplete understanding of the barriers and successes of these initiatives.

Furthermore, the study’s narrative approach may be subject to selection and interpretive biases. For instance, the emphasis on well-documented and large-scale programs, such as NSOAPs and the GNI, may overlook smaller, community-driven projects that also contribute significantly to neurosurgical advancements. Finally, the dynamic and complex nature of health policy development and implementation in LMICs may not have been fully captured in this review. Rapidly evolving political, economic, and cultural factors influence the sustainability and scalability of these initiatives, making it difficult to provide a definitive assessment of their long-term impact. These limitations highlight the need for ongoing research and data collection to better understand and address the challenges faced in achieving equitable neurosurgical care globally. While this review provides valuable insights, the findings should be interpreted with caution and contextualized within the broader scope of global health policy and advocacy efforts.

## Conclusion

The trajectory of global neurosurgery advocacy and policy has been marked by significant achievements and ongoing challenges. Collaborative efforts and innovative strategies are pivotal in reshaping the landscape, ultimately bridging the gap between regions and socioeconomic strata and ensuring that neurosurgical care is a universal right, not a privilege. As the divide between LMICs and HICs persists, the field’s evolution has been defined by both achievements and obstacles. Advocacy and policy, acting as catalysts, have spurred advancements that transcend geographic and economic boundaries. Yet, this path is riddled with challenges. Scarce resources, limited awareness, intricate political dynamics, and fragmented collaboration have impeded the pace of progress. But much progress is being made. Collaborations between healthcare, technology, and government sectors are leading to data-driven approaches in global health diplomacy, which are being amplified by digital platforms and AI-driven analytics. The patient’s voice is becoming more prominent in advocacy efforts; as interdisciplinary partnerships shape comprehensive approaches. In particular, a strong commitment to reducing global health inequalities is driving policies toward universal access to neurosurgical care. The pursuit of equitable neurosurgical care is an ongoing endeavor that requires collective resolve. As policies evolve and advocacy strategies adapt, the vision of accessible, safe, and affordable neurosurgical care for all becomes increasingly clear. Through innovative approaches, the global neurosurgery community can chart a course toward a future where no person is left behind, regardless of their location or economic situation.

## Data Availability

No new data was generated.
